# Multicriteria decision analysis in the Italian healthcare context: a review of applications and methodological approaches

**DOI:** 10.3389/fpubh.2026.1839935

**Published:** 2026-05-15

**Authors:** Riccardo Mercati, Chiara Vassallo, Francesca Donnaloja

**Affiliations:** IQVIA Solutions Italy S.r.l., Milan, Italy

**Keywords:** health policy, health technology assessment, healthcare decision-making, Italy, MCDA, multicriteria decision analysis, preference elicitation, stakeholder involvement

## Abstract

Multicriteria Decision Analysis (MCDA) has gained increasing relevance in healthcare as an approach for evaluating options across multiple criteria. This study aims to provide an overview of MCDA applications in Italy, examining their decision contexts, the preference elicitation methods employed, and the stakeholder groups involved. A literature review was conducted across PubMed and Google Scholar (searches up to November 2025), supplemented by Italian studies identified in an international systematic review. For each study, data were collected on publication year, decision context, preference elicitation methods, and stakeholder involvement. Nineteen studies met the eligibility criteria, 16 of which were published after 2017. Most applications concerned priority-setting decisions related to coverage, reimbursement or funding of healthcare interventions, particularly the evaluation of drugs, therapeutic products, and other health technologies. Preference elicitation methods were predominantly compositional, with direct rating and the Analytic Hierarchy Process being the most frequently applied techniques; decompositional approaches were rarely used. Approximately half of the studies implemented a multi-stakeholder approach. Clinicians were the most frequently included group, followed by healthcare providers, while other stakeholder categories, including patients, were less commonly involved. The use of MCDA in Italy is progressively increasing and generally aligns with global trends. However, important opportunities for further development remain. These include extending MCDA applications to earlier stages of the technology lifecycle, adopting more rigorous and robust preference elicitation techniques, and strengthening its multi-stakeholder orientation, particularly by enhancing patient involvement, in order to improve the methodological quality and policy relevance of future MCDA applications in the Italian context.

## 1 Introduction

Healthcare decision making encompasses a wide range of choices, including the adoption of health technologies, clinical pathways, service delivery models, and public health interventions (1, 2). Across these contexts, decisions require balancing multiple dimensions of value that reflect not only clinical outcomes and safety, but also economic, ethical, organizational, and societal considerations (1, 2). In addition, healthcare decisions typically involve multiple stakeholders, such as patients and caregivers, clinicians, healthcare providers, payers, and policy makers, each of whom may place different emphasis on objectives such as clinical benefit, safety, affordability, equity, feasibility of implementation, or system sustainability (1, 3).

While economic evaluation has traditionally focused on costs, efficiency, and health gains, these approaches are generally not designed to explicitly consider multiple value dimensions simultaneously or to assign relative priorities across them (4, 5). As a result, they may be limited in their ability to reflect and balance diverse stakeholder perspectives, particularly in decision contexts involving trade-offs that go beyond the maximization of aggregate health and economic outcomes (4, 5). Thus, these limitations underscore the need for complementary evaluation approaches that can more adequately address the complexity and plurality of values characterizing healthcare decision-making.

The need for more multidimensional, multi-stakeholder, and transparent evaluation processes has been further reinforced at the policy level by the recent European Health Technology Assessment Regulation (EU) 2021/2282, which aims to strengthen cooperation across Member States and promote consistent and inclusive assessment processes to support healthcare decision making at the European level (6).

In this context, Multicriteria Decision Analysis (MCDA) has emerged over recent years as a methodological approach of growing interest in the healthcare literature to support complex decision making characterized by multiple value dimensions and stakeholder perspectives (7–9). MCDA refers to a family of structured decision-support methods designed to enable the explicit comparison of alternative options, such as health technologies, interventions, or service models, across multiple, potentially conflicting criteria, using transparent and systematic procedures (7, 8). By decomposing decisions into clearly defined criteria and making trade-offs explicit, MCDA provides a framework that is specifically suited to contexts in which value cannot be adequately summarized by a single outcome measure.

In order to support highly structured decision-making processes, MCDA involves the identification of relevant evaluation criteria, the scoring of alternatives according to their performance on each criterion, and the weighting of criteria to reflect their relative importance in the decision-making process (7, 8, 10, 11). Scoring captures stakeholders’ preferences regarding differences in performance within each criterion (e.g., clinical effectiveness, costs, organizational impact), whereas weighting reflects the relative priority assigned to each criterion when trade-offs are required (7, 8). Table 1 and Supplementary Table 1 provide an overview of the main scoring and weighting techniques used in MCDA applications in healthcare (9), summarizing their key characteristics as well as their respective advantages and limitations for both compositional approaches, where respondents directly assess the importance of criteria or the performance of alternatives on individual criteria, and decompositional approaches, which infer preferences indirectly by analyzing choices among hypothetical alternatives combining multiple criteria (7, 8).

**Table 1:** Characteristics of preference elicitation methods for MCDA.

**Characteristic**	**Compositional methods**					**Decompositional methods**	
	**AHP**	**Direct rating**	**MACBETH**	**Swing weighting**	**Value functions**	**DCE**	**PAPRIKA**
Applicability to both scoring and weighting	✔✔	✔✔	✔✔	—	—	✔✔	✔✔
Scoring	✔✔	✔✔	✔✔	—	✔✔	✔✔	✔✔
Weighting	✔✔	✔✔	✔✔	✔✔	—	✔✔	✔✔
Ease of research methods	✔	✔ ✔	✔	✔	✔	—	—
Ease of study design	✔	✔✔	✔	✔	✔	—	—
Ease of data analysis	✔	✔✔	✔	✔✔	✔✔	—	—
Ease of respondent involvement	✔	✔✔	✔	✔	✔	—	✔
Low cognitive burden	✔	✔✔	—	—	—	✔	✔✔
Limited sample size required	✔✔	✔✔	✔✔	✔✔	✔✔	—	✔
Robustness of results	—	—	✔	✔	✔	✔✔	✔
Realistic choice tasks	—	—	—	—	—	✔✔	✔
Ability to capture trade-offs	—	—	✔	✔	—	✔✔	✔
Scores respect interval properties	—	—	—	*n.a.*	✔	✔✔	✔
Weights are scaling constants	—	—	✔✔	✔✔	*n.a.*	✔✔	✔✔

**—** = The method does not respect the characteristic; ✔ = The method partially respects the characteristic; ✔✔ = The method fully respects the characteristic; n.a. = The characteristic is not applicable, as the method is not intended for scoring/weighting. Author-developed conceptual synthesis based on the published literature (8, 11, 40–46). AHP = Analytic Hierarchy Process; DCE = Discrete Choice Experiment; MACBETH = Measuring Attractiveness by a Categorical Based Evaluation Technique; PAPRIKA = Potentially All Pairwise Rankings of All Possible Alternatives.

Although the literature has well documented the growing international use of MCDA in healthcare decision-making (9), highlighting substantial heterogeneity in terms of decision contexts, stakeholder involvement, and methodological approaches for preference elicitation (9), existing reviews rarely provide structured, country-level syntheses of how MCDA has been implemented within national healthcare settings. Overall, the available evidence suggests further heterogeneity in MCDA applications across countries, likely reflecting differences in institutional settings, decision-making objectives, and levels of implementation (9, 12, 13). For example, among European countries, the literature suggests that MCDA adoption in France has remained at an early stage, with its formal role in national HTA still debated (14), whereas in Spain MCDA appears to have been applied more extensively, particularly at the regional level, to support prioritization, reimbursement, and early access decisions (15–17).

Against this background, evidence specifically addressing how MCDA has been applied within the Italian healthcare context is still scarce. Building on the analytical perspective adopted in the international review from Gongora-Salazar et al. (9), this study analyzes MCDA applications involving the Italian healthcare context, focusing on decision contexts, preference elicitation methods, and stakeholder involvement, and discusses their alignment with international trends.

## 2 Materials and methods

### 2.1 Study design

The design of the literature search, including search strategies, eligibility and exclusion criteria, and data extraction procedures, was guided by the international literature review by Góngora-Salazar et al. (9) and adapted to address the specific objectives of the present analysis, with a focus on applications of MCDA within the Italian healthcare context.

### 2.2 Identification of studies

Relevant published literature was identified through targeted searches in PubMed and Google Scholar. In line with the literature (9), Google Scholar searches were restricted to the first 100 results.

The search strategy elaborated on methodological terms related used Gongora-Salazar et al. (9) to identify MCDA applications and preference elicitation methods (including variations of “MCDA” and related approaches), and was integrated with the addition of geographical identifiers to capture studies referring to the Italian context (e.g., keywords beginning with “Ital*”). The complete search strings are reported in Supplementary Table 2. Searches were conducted up to November 2025, with no restrictions on publication year, thereby extending the time horizon beyond that considered in the international review (9), which included studies published up to 2020.

In addition to database searches, citation searching was performed on the reference list of the international systematic review by Gongora-Salazar et al. (9) to identify additional relevant publications not captured through the predefined search strings.

All reviewers contributed to the development of the search strategy.

### 2.3 Screening and inclusion of studies

Study selection followed predefined inclusion and exclusion criteria that were largely consistent with those applied by Gongora-Salazar et al. (9); however, minor adaptations were implemented to ensure that the language and geographical scope of the retrieved studies appropriately reflected the aims of the present review. Studies were included if they:

1. Were available as full-text publications in English or Italian;
2. Reported an empirical application of MCDA to support healthcare decision making including the Italian context;
3. Provided a description of the MCDA methodology applied.

Studies were excluded if they:

1. Did not apply MCDA;
2. Did not implement the methodological steps outlined in the ISPOR MCDA Good Practices (7, 8);
3. Did not aim to inform healthcare decision making in Italy.

Titles and abstracts retrieved through the searches were screened against the eligibility criteria, and duplicate records as well as studies clearly not meeting the inclusion criteria were excluded during this first screening phase. Full-texts were subsequently analyzed for records considered potentially eligible and were assessed in detail to confirm inclusion.

Title and abstract screening, as well as full-text assessment, were initially performed by one reviewer. A second reviewer independently assessed all studies selected for inclusion. Any uncertainties or disagreements were resolved through discussion among all reviewers.

### 2.4 Information extraction

For each included study, data were extracted on the following dimensions:

- Year of publication;
- Decision context, defined in terms of the goal and setting of the decision. Contexts were classified drawing on classifications used in previous literature (which used priority setting, clinical decision making, regulatory decision making – benefit–risk assessment, and planning and research and development as main categories) (9, 18), and adapted to reflect the Italian context where appropriate;
- Scoring and weighting method, whose main definitions are detailed in Supplementary Table 1;
- Type of stakeholders involved in the MCDA process.

Information extraction was performed independently by two reviewers. All reviewers jointly discussed issues related to classification and categorization of studies until consensus was reached. In particular, with regard to preference elicitation methods, when the specific scoring or weighting techniques were not explicitly named, the applied method was, whenever feasible, inferred from the methodological details reported in the publication.

## 3 Results

**Figure 1:**
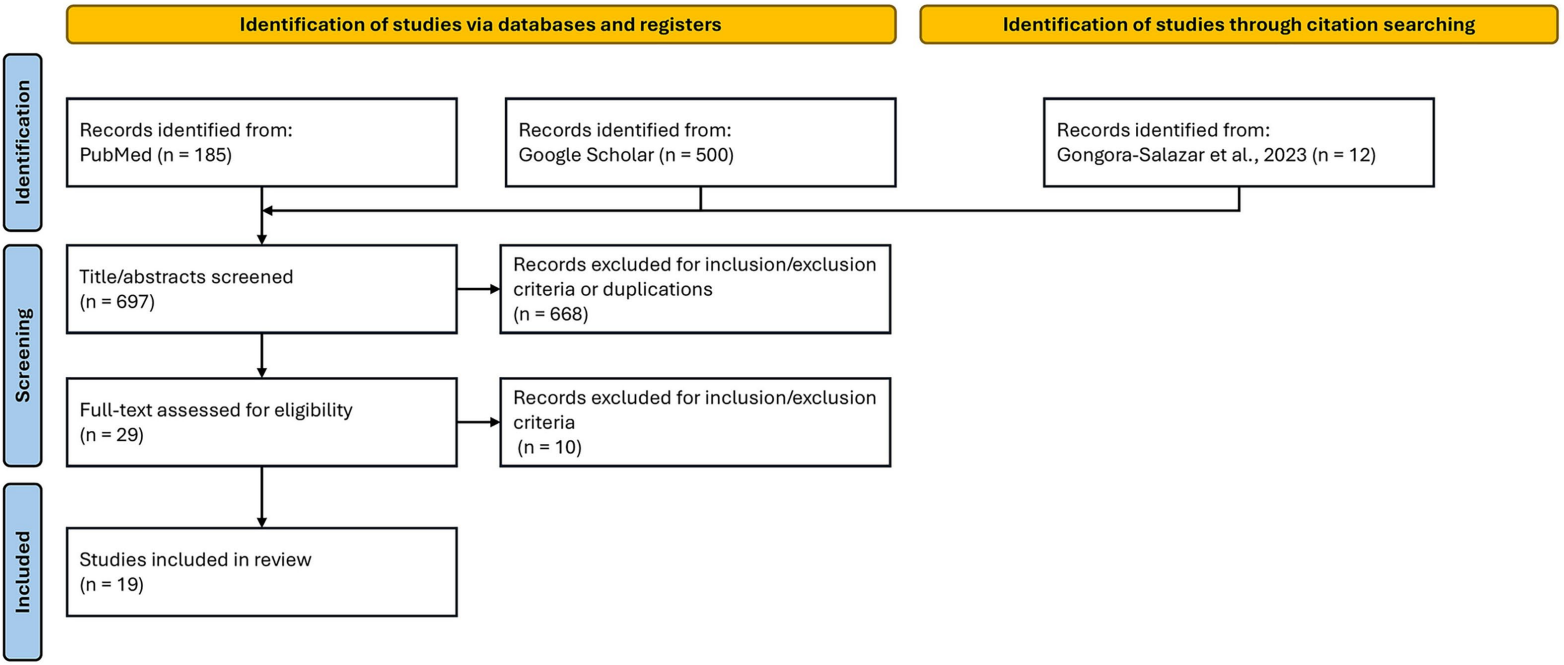
Study selection flowchart. Records identified up to November 7, 2025.

As reported in Figure 1, a total of 697 titles and abstracts were screened. Of these, 29 articles were retrieved for full-text assessment. Ultimately, 19 studies met the eligibility criteria and were included in the review (19–37). Of the studies included in the analysis, the majority (*N* = 13, 68.4%) were published between 2017 and 2020. The remaining studies were evenly distributed across earlier and later periods, with 3 (15.8%) published before 2017, the earliest dating back to 2009, and 3 (15.8%) after 2020, with the latest dating to 2025.

### 3.1 Decisional contexts

Table 2 summarizes the decision-making contexts in which MCDA has been applied in studies that include the Italian healthcare setting. The majority of the studies concerned priority-setting decisions (*N* = 15, 78.9%), predominantly focusing on coverage, reimbursement or funding decisions for healthcare interventions (*N* = 14, 73.7%). Within this category, most applications involved drugs and therapeutic products (*N* = 7, 36.8%), followed by other health technologies (*N* = 6, 31.6%), predominantly including diagnostic and medical devices. Only one study (5.3%) examined prioritization of patients access to healthcare in contexts marked by constrained resources and limited inpatient capacity.

**Table 2:** Decisional contexts of MCDA applications.

Decisional context	Number of publications (%)
Priority setting	15 (78.9%)
Coverage, reimbursement or funding of healthcare interventions	14 (73.7%)
Drugs and therapeutic products	7 (36.8%)
Other health technologies	6 (31.6%)
Health policies	1 (5.3%)
Patients to access healthcare	1 (5.3%)
Planning, logistical, and organizational decisions in hospital setting	2 (10.5%)
Clinical decision making for drug prescription	1 (5.3%)
Benefit–risk analysis for vaccines	1 (5.3%)
Total	19 (100.0%)

Other decisional domains were less represented, with studies addressing planning, logistical and organizational decisions in the hospital setting (*N* = 2, 10.5%), clinical decision-making for drug prescribing (*N* = 1, 5.3%) and benefit–risk assessment for vaccines (*N* = 1, 5.3%). No studies applied MCDA to early-stage product development, indicating that these areas remain entirely unexplored within the Italian context.

### 3.2 Preference elicitation methods

**Figure 2:**
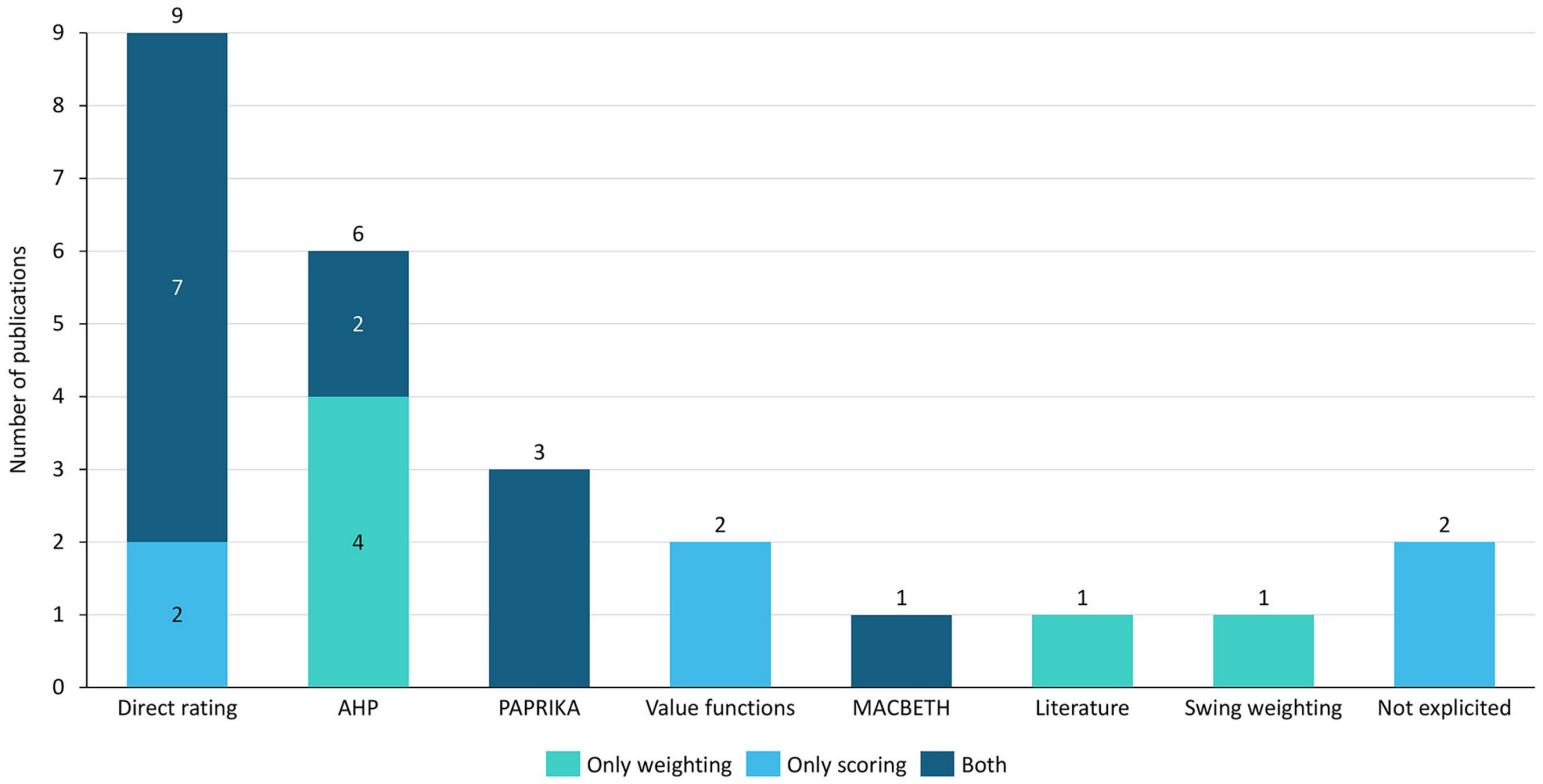
Scoring and weighting methods used in analyzed publications. AHP = Analytic Hierarchy Process; MACBETH = Measuring Attractiveness by a Categorical Based Evaluation Technique; PAPRIKA = Potentially All Pairwise Rankings of All Possible Alternatives.

Figure 2 summarizes the preference elicitation methods adopted for weighting and scoring across the included studies. In most cases (*N* = 13; 68.4%), the same methodological approach was used for both weighting and scoring; however, in two studies (10.5%) the scoring method could not be clearly identified from the available information. Overall, compositional approaches were predominant, whereas only three studies (15.8%) relied on decompositional methods.

Among compositional techniques, direct rating was the most frequently applied method (*N* = 9, 47.4%) and was commonly used for both weighting and scoring. The Analytic Hierarchy Process (AHP) represented the second most frequently adopted approach (*N* = 6, 31.6%), mainly employed for weighting purposes. Other compositional methods, including value functions (*N* = 2, 10.5%), Measuring Attractiveness by a Categorical-Based Evaluation Technique (MACBETH) (*N* = 1, 5.3%), and swing weighting (*N* = 1, 5.3%), were used only sporadically.

By contrast, decompositional methods were rarely employed. All studies adopting a decompositional approach relied on Potentially All Pairwise Rankings of All Possible Alternatives (PAPRIKA) (*N* = 3, 15.8%), while no study applied Discrete Choice Experiments (DCEs).

### 3.3 Stakeholder involvement

Approximately half of the studies (*N* = 10, 52.6%) adopted a multi-stakeholder approach, involving more than one category of stakeholders, while one study (5.3%) did not provide explicit information on the stakeholders involved.

As shown in Supplementary Figure 1, clinicians were the most frequently included group, engaged in the majority of the studies (*N* = 11, 57.9%). They were followed by healthcare providers (*N* = 8, 42.1%), such as hospital health directors and public health specialists. Methodologists, patients, and representatives from health authorities were engaged to a moderate extent (*N* = 5, 26.3% each). A smaller number of studies involved other types of stakeholders. When considering studies that reported the involvement of a single stakeholder group (*N* = 8, 42.1%), most included clinicians only (*N* = 5), followed by healthcare providers (*N* = 2) and patients (*N* = 1). Overall, this distribution mirrors the pattern observed across the full set of included studies.

## 4 Discussion

Multicriteria Decision Analysis has emerged in the international literature as a structured methodological framework that supports complex decision-making by systematically comparing alternative options across multiple and often conflicting criteria (7–9, 11). Its consolidation as a strategic tool in healthcare stems from its ability to combine methodological rigor with the growing need for inclusiveness and flexibility in increasingly complex decision environments (38).

Since 2010, the international literature has reported a marked increase in healthcare-related MCDA studies. This trend is largely attributed to the expansion of Health Technology Assessment (HTA) agencies across Europe, mounting pressures on governments to justify investment and authorization decisions, and the broader application of MCDA to new decision-making contexts (9, 39). In Italy, although the number of applications remains relatively limited, the use of MCDA has expanded over the past decade. Most applications were published after the release of the ISPOR Good Practice guidelines (7, 8), suggesting that these recommendations likely stimulated their development and reflecting a growing interest in formalizing decision-making processes within the national healthcare context.

Regarding the applicability of MCDA, international evidence highlights its use across a broad spectrum of decision-making contexts. Approximately half of all applications relate to priority-setting, most commonly in decisions concerning coverage, reimbursement, and the allocation of funding for healthcare interventions. The remaining applications span other decision contexts, including clinical decision making, regulatory assessments, and research and development processes (9). Overall, this breadth of applications reflects the adaptability of MCDA across different stages of the healthcare decision-making lifecycle (9). As a result of our analysis, a partially overlapping pattern emerges in Italy, where priority-setting also represents the predominant decision context, while the remaining categories show only limited uptake. Notably, no Italian studies were identified in the area of research and development, indicating that current applications are predominantly concentrated in the final stages of the HTA process, where decisions on coverage, reimbursement and prioritization are made. This gap also suggests that earlier phases of the product lifecycle may represent a promising yet unexplored domain for future MCDA development and implementation in Italy.

Beyond the decision-making contexts in which MCDA is applied, the selection of preference elicitation methods represents a critical determinant of the rigor, validity, and reproducibility of MCDA results (7–9, 38). However, both Italian and international MCDA applications frequently lack clear and accurate reporting on the scoring and weighting techniques employed, as well as explicit justifications for methodological choices, reflecting limited standardization across classifications and approaches (9). In line with global evidence, most Italian studies rely on compositional approaches, which are generally easier to implement and require smaller sample sizes (8, 11, 40–46). Within this group, direct rating methods are the most frequently adopted, followed by the Analytic Hierarchy Process (AHP), a pattern consistent with the preference for approaches that are relatively straightforward to administer. Nevertheless, even within compositional approaches, several elicitation techniques offer more rigorous theoretical foundations and yield more robust results than commonly used methods such as direct rating and AHP (8, 9, 11, 40–43, 46). Conversely, decompositional approaches, particularly DCEs, which have strong inferential properties, remain less commonly used in MCDA studies, likely due to their higher design complexity, greater analytical requirements, and the need for larger respondent samples (8, 9, 11, 40–46). In this context, the selection of preference elicitation methods should, in the first instance, be guided by the objectives of the research and by the alignment between the chosen approach and feasibility constraints related to time, resources, or sample availability. Within this framework, when the research question is compatible with the use of more analytically robust approaches and feasibility conditions allow, the adoption of more theoretically grounded elicitation methods may be preferable, as they can enhance the robustness, interpretability and generalizability of MCDA outcomes.

Moreover, a key strength of MCDA lies in its ability to integrate the perspectives of diverse stakeholder groups through a structured approach, an element increasingly regarded as fundamental for robust, transparent, and socially legitimate decision-making in healthcare (7, 8, 47). While international evidence indicates that nearly half of MCDA studies do not clearly report stakeholder involvement (9), the Italian literature generally provides more explicit documentation of stakeholder categories, thereby enhancing transparency in the inclusion process. Despite this positive trend, around half of Italian MCDA applications do not employ a multi-stakeholder framework, representing a limitation in decision contexts that would benefit from broader stakeholder involvement. Furthermore, although MCDA applications commonly include at least one category of clinical or institutional decision makers, consistent with international evidence showing that healthcare providers and subject-matter experts are the most frequently included stakeholder groups (9), patients were involved in only about one quarter of the studies reviewed. Greater patient inclusion would be advisable, where feasible and relevant, particularly in light of the forthcoming European HTA Regulation (EU 2021/2282) (6) which, effective January 2025, requires the structured and active participation of patient organizations in Joint Clinical Assessments (JCAs). International literature consistently emphasizes the importance of involving patients not only in regulatory processes but also in earlier phases of the decision-making continuum, including the identification of unmet needs, the selection of relevant endpoints, benefit–risk assessments, and post-marketing evaluations (48). Expanding patient engagement within Italian MCDA practice would therefore strengthen the legitimacy, multidimensionality, and contextual relevance of decision frameworks, aligning national practice with evolving international standards and expectations.

In conclusion, MCDA applications to support complex healthcare decisions have expanded in Italy over the past decade, particularly in priority-setting and HTA-related processes, although the overall number of publications remains limited. Despite this growth, opportunities for improvement remain, in line with international trends. Even though MCDA offers substantial methodological potential, most applications continue to rely on simpler and less robust elicitation approaches, largely due to considerations of feasibility. Moreover, the adoption of a multi-stakeholder perspective and the inclusion of patient voices, when relevant to the decision context, could be further strengthened, especially as patient involvement is expected to become a formal requirement under the new European HTA Regulation (6). Thus, broadening the use of MCDA to earlier stages of the technology lifecycle, adopting more rigorous elicitation methods where feasible, and extending engagement to a more diverse set of stakeholders could meaningfully enhance the quality and impact of healthcare decision-making processes in Italy.
