# Supplementary material for: Multicriteria decision analysis in the Italian healthcare context: a review of applications and methodological approaches
Source: Front Public Health. 2026 May 15;14:1839935. doi: 10.3389/fpubh.2026.1839935 (PMC13219325; doi:10.3389/fpubh.2026.1839935)
Supplement: Supplementary file 1 [file Data_Sheet_1.docx]

Supplementary Material

# Supplementary Figures and Tables

Supplementary table 1 Summary of scoring and weighting procedures in preference elicitation methods for MCDA

| **Methodology** | | **Elicitation phases** | |
| --- | --- | --- | --- |
|  |  | **Scoring** | **Weighting** |
| **Compositional** | **AHP** | - Respondents perform pairwise comparisons of alternatives for each criterion - Each comparison uses a relative importance scale (e.g., equal to extreme importance) - Scores for alternatives are calculated by normalizing ratio values from these comparisons using eigenvalue analysis | - Criteria are structured hierarchically to break down the decision problem - Each level of the hierarchy can be analyzed as a separate decision problem - Respondents compare criteria pairwise using the same importance scale - Weights are derived from ratios using eigenvalue analysis |
|  | **Direct rating** | - Rating scales: for each criterion, each alternative is assigned a rating on a 0-100 (or equivalent) scale. This rating reflects its performance compared to the other alternatives - Point allocation: for each criterion, a fixed number of points (commonly 100) is distributed among the alternatives based on their relative performance on the criterion | - Rating scales: each criterion is assigned a rating on a 0-100 (or equivalent) scale. This rating reflects its importance compared to the other criteria - Point allocation: A fixed number of points (commonly 100) is distributed among the criteria based on their relative importance |
|  | **MACBETH** | - Respondents perform pairwise comparisons of alternatives for each criterion - The difference in attractiveness between alternatives is elicited through qualitative judgements - Judgments are converted into numerical scales through an iterative process | - Respondents rank criteria based on their swings (i.e., the range from the worst and the best outcome within each criterion) and provide qualitative judgments about the desirability of the swings - Then, respondents make pairwise comparisons across criteria, judging how much more desirable one swing is over another - Weights are derived by converting qualitative judgments into a consistent numerical scale |
|  | **Swing weighting** | Not used | - First, criteria are ranked based on the importance of their swings (i.e., the range from worst to best outcome within each criterion) - Then, a score of 100 is assigned to the top-ranked swing and rate the others relative to it (0–100) |
|  | **Value function** | - Once the swings (i.e., the range from the worst and the best outcome within each criterion) for each criterion are defined, value functions are constructed to translate performance levels into scores on a 0–100 scale - If linear functions are used, the relationship between performance and score is proportional - Alternatively, non-linear functions can be built using methods like bisection (i.e., respondents identify the performance level corresponding to a score of 50) or the difference method (i.e., where they assign a 0-100 score to the midpoint of the performance range). In both cases, the swing endpoints define scores of 0 and 100, and the process is iterated over subranges to refine the value function | Not used |
| **Decompositional** | **DCE** | - Respondents are presented with a series of choice sets, each composed of two (or more) hypothetical scenarios - Each hypothetical scenario represents a specific combination of criteria and levels - For each choice task, respondents indicate their preferred scenario - Weights and scores are calculated across all participants using regression models | |
|  | **PAPRIKA** | - Respondents make choices based on pairwise comparisons between scenarios defined on a subset of criteria (two or more) - Each comparison involves a trade-off between criteria - After each choice, the method applies transitivity to infer additional preferences and eliminate all possible scenarios that are logically dominated by previous selections - Weights and scores are derived using linear programming based on all pairwise preferences | |

Acronyms: Acronyms: AHP = Analytic Hierarchy Process; DCE = Discrete Choice Experiment; MACBETH = Measuring Attractiveness by a Categorical Based Evaluation Technique; PAPRIKA = Potentially All Pairwise Rankings of All Possible Alternatives
Notes: Authors’ descriptive adaptation and synthesis of the published literature [1–7]

Supplementary table 2 Search terms

| **Search engine** | **Search terms** |
| --- | --- |
| PubMed | (MCDA[Title/Abstract]) AND (Ital*[Title/Abstract]) |
|  | (Multi criteria decision analysis[Title/Abstract]) AND (Ital*[Title/Abstract]) |
|  | (Multi criteri* decision* [Title/Abstract]) AND (Ital*[Title/Abstract]) |
|  | (Multicriteria decision analysis[Title/Abstract]) AND (Ital*[Title/Abstract]) |
|  | (Multicriteri* decision* [Title/Abstract]) AND (Ital*[Title/Abstract]) |
|  | (Multiple criteria decision analysis[Title/Abstract]) AND (Ital*[Title/Abstract]) |
|  | (Multiple criteri* decision* [Title/Abstract]) AND (Ital*[Title/Abstract]) |
|  | (MCDM[Title/Abstract]) AND (Ital*[Title/Abstract]) |
|  | (Multi-attribute utility[Title/Abstract]) AND (Ital*[Title/Abstract]) |
|  | (Multi-objective optim*[Title/Abstract]) AND (Ital*[Title/Abstract]) |
|  | (Weighted product method[Title/Abstract]) AND (Ital*[Title/Abstract]) |
|  | (Goal programming[Title/Abstract]) AND (Ital*[Title/Abstract]) |
|  | ((Analytical hierarchy process[Title/Abstract]) OR (AHP[Title/Abstract])) AND (Ital*[Title/Abstract]) |
|  | ((Analytic network process[Title/Abstract]) OR (ANP[Title/Abstract])) AND (Ital*[Title/Abstract]) |
|  | ((Measuring attractiveness by a categorical based evaluation technique [Title/Abstract]) OR (MACBETH[Title/Abstract])) AND (Ital*[Title/Abstract]) |
|  | ((Technique for order preference by similarity to ideal solution [Title/Abstract]) OR (TOPSIS[Title/Abstract])) AND (Ital*[Title/Abstract]) |
|  | ((Preference ranking organization method of enrichment evaluation [Title/Abstract]) OR (PROMETHEE[Title/Abstract])) AND (Ital*[Title/Abstract]) |
|  | (Elimination and choice expressing reality[Title/Abstract]) AND (Ital*[Title/Abstract]) |
| Google Scholar - first 100 results | “Multi-criteria decision” health "Italy" |
|  | "Multicriteria decision” health “Italy" |
|  | “Multiple criteria decision” health "Italy" |
|  | “Multicriteria analysis” health "Italy" |
|  | “MCDA” health "Italy" |

**Supplementary figure 1 Stakeholders included in analyzed publications**

**
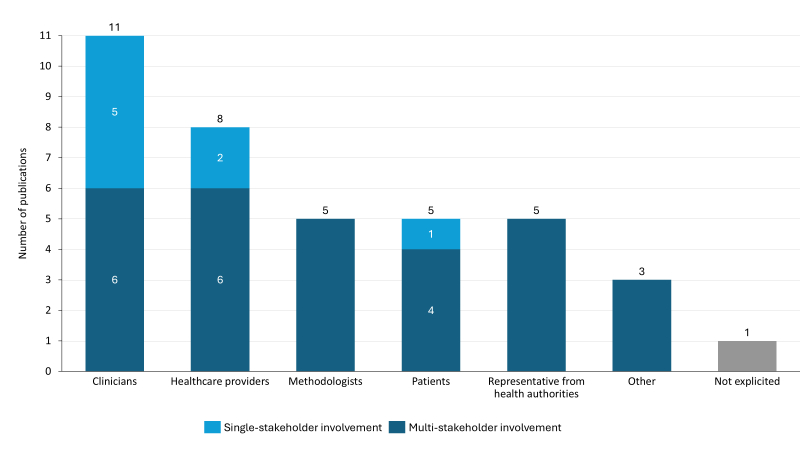
**

# Bibliography

1. Hansen P, Devlin N. Multi-Criteria Decision Analysis (MCDA) in Healthcare Decision-Making. In: Oxford Research Encyclopedia of Economics and Finance [Internet]. 2019 [citato 20 marzo 2026]. Disponibile su: https://oxfordre.com/economics/display/10.1093/acrefore/9780190625979.001.0001/acrefore-9780190625979-e-98 doi:10.1093/acrefore/9780190625979.013.98

2. Thokala P, Devlin N, Marsh K, Baltussen R, Boysen M, Kalo Z, et al. Multiple Criteria Decision Analysis for Health Care Decision Making—An Introduction: Report 1 of the ISPOR MCDA Emerging Good Practices Task Force. Value Health. 1 gennaio 2016;19(1):1–13. doi:10.1016/j.jval.2015.12.003 PubMed PMID: 26797229.

3. Gongora-Salazar P, Rocks S, Fahr P, Rivero-Arias O, Tsiachristas A. The Use of Multicriteria Decision Analysis to Support Decision Making in Healthcare: An Updated Systematic Literature Review. Value Health. 1 maggio 2023;26(5):780–90. doi:10.1016/j.jval.2022.11.007 PubMed PMID: 36436791.

4. consortium TP. PREFER Recommendations - Why, when and how to assess and use patient preferences in medical product decision-making [Internet]. 19 aprile 2022. doi:10.5281/zenodo.6592304

5. Marsh K, Thokala P, Mühlbacher A, Lanitis T. Incorporating Preferences and Priorities into MCDA: Selecting an Appropriate Scoring and Weighting Technique. In: Marsh K, Goetghebeur M, Thokala P, Baltussen R, curatori. Multi-Criteria Decision Analysis to Support Healthcare Decisions [Internet]. Cham: Springer International Publishing; 2017 [citato 20 marzo 2026]. p. 47–66. Disponibile su: https://doi.org/10.1007/978-3-319-47540-0_4 doi:10.1007/978-3-319-47540-0_4

6. Beyer A, Fasolo B, Costa J, Bana e Costa C. Application of MACBETH to the elicitation of preferences for treatment outcomes. 2014.

7. Bana E Costa CA, De Corte JM, Vansnick JC. Macbeth. Int J Inf Technol Decis Mak. marzo 2012;11(02):359–87. doi:10.1142/S0219622012400068
